# Rapid and sustained response to JAK inhibition in a child with severe MDA5 + juvenile dermatomyositis

**DOI:** 10.1186/s12969-023-00894-9

**Published:** 2023-09-19

**Authors:** Timmy Strauss, Claudia Günther, Anja Schnabel, Christine Wolf, Gabriele Hahn, Min Ae Lee-Kirsch, Normi Brück

**Affiliations:** 1 Faculty of Medicine, Department of Pediatrics, Pediatric Rheumatology and Immunology, University Hospital Carl Gustav Carus, Technische Universität Dresden, Dresden, Germany; 2 Faculty of Medicine, Department of Dermatology, University Hospital Carl Gustav Carus, Technische Universität Dresden, Dresden, Germany; 3 Faculty of Medicine, Department of Pediatrics, Molecular Pediatrics, University Hospital Carl Gustav Carus, Technische Universität Dresden, Dresden, Germany; 4 Faculty of Medicine, Department of Radiology, Pediatric Radiology, University Hospital Carl Gustav Carus, Technische Universität Dresden, Dresden, Germany

**Keywords:** Juvenile dermatomyositis, MDA5 autoantibody, Janus kinase inhibition

## Abstract

**Background:**

Juvenile dermatomyositis (jDM) is the most common idiopathic inflammatory myopathy of childhood. Amyopathic or hypomyopathic courses have been described.

**Case presentation:**

We present the case of a 4-year-old patient with MDA5 antibody positive jDM and interstitial lung disease. In our patient, typical symptoms of jDM with classical skin lesions, arthritis, proximal muscle weakness, and ulcerative calcifications were observed. Due to the severity of the disease and the pulmonary changes, therapy with the Janus kinase (JAK) inhibitor ruxolitinib was added to the therapy with corticosteroids, intravenous immunoglobulins (IVIG) and hydroxychloroquine leading to a fast and sustained remission.

**Conclusion:**

While there is growing evidence that JAK inhibition is a promising therapeutic option in jDM our case report shows that this approach may also be effective in MDA5-positive jDM with high risk features.

**Supplementary Information:**

The online version contains supplementary material available at 10.1186/s12969-023-00894-9.

## Background: MDA5-positive juvenile dermatomyositis

Juvenile dermatomyositis (jDM) is the most common idiopathic inflammatory myopathy of childhood [1]. This systemic autoimmune disease is associated with typical changes in the skin, vasculopathy, and muscle weakness that is usually trunk accentuated [2]. The exact etiology of jDM is still unclear. It is discussed that based on a genetic predisposition, external environmental factors trigger an autoimmune response [3]. In the pathogenesis of vasculopathy in jDM, activation of type I interferon-induced genes seems to play an important role [4, 5]. In 60–90% of patients with jDM, myositis-specific antibodies, such as anti-TIF 1-γ (p155), anti-NXP2/(p140/MJ), anti-MDA5, as well as myositis-associated antibodies, such as anti-La (‘SSB’), anti-Ro (‘SSA’), and anti-Sm, can be detected and helpful in establishing the diagnosis [6, 7]. *Melanoma differentiation-associated protein 5* (MDA5) is physiologically involved as a pattern recognition receptor in the recognition of viral nucleic acid sequences. Here, the ongoing signaling cascade leads to activation of the type I interferon response [8, 9]. It has been shown that different autoantibodies are associated with different clinical phenotypes. MDA5 autoantibodies are associated with an increased risk of skin ulceration, arthritis, interstitial lung disease (ILD), as well as an amyopathic or hypomyopathic course in jDM [6]. Sontheimer et al. define hypomyopathic dermatomyositis as “dermatomyositis-specific skin disease and no clinical evidence of muscle disease (i.e., weakness) that are found to have subclinical evidence of myositis upon laboratory, electrophysiological and/or radiological evaluation” [10]. A review from 2022 [11] reported that up to 80% of patients with clinically amyopathic dermatomyositis can develop ILD. If MDA5 autoantibodies are present, the association with ILD is even higher (up to 95%). The ILD in MDA5 autoantibody positive jDM is critical due to its treatment refractory course and high mortality rates (6-month mortality up to 50%). Therapy of jDM is based on the administration of glucocorticoids, intravenous immunoglobulins (IVIG), and cDMARDs (such as methotrexate, mycophenolate mofetil, and azathioprine). In severe courses or refractory disease, drugs such as rituximab, TNF-alpha inhibitors, or cyclophosphamide are used [1, 12, 13]. Given the crucial role of the JAK STAT pathway in the pathophysiology of the disease, JAK inhibitors represent a reasonable therapeutic option in jDM. Several case reports describe a positive effect of JAK inhibition (ruxolitinib, tofacitinib, baricitinib) in refractory jDM [14–16].

## Case presentation

**Fig. 1:**
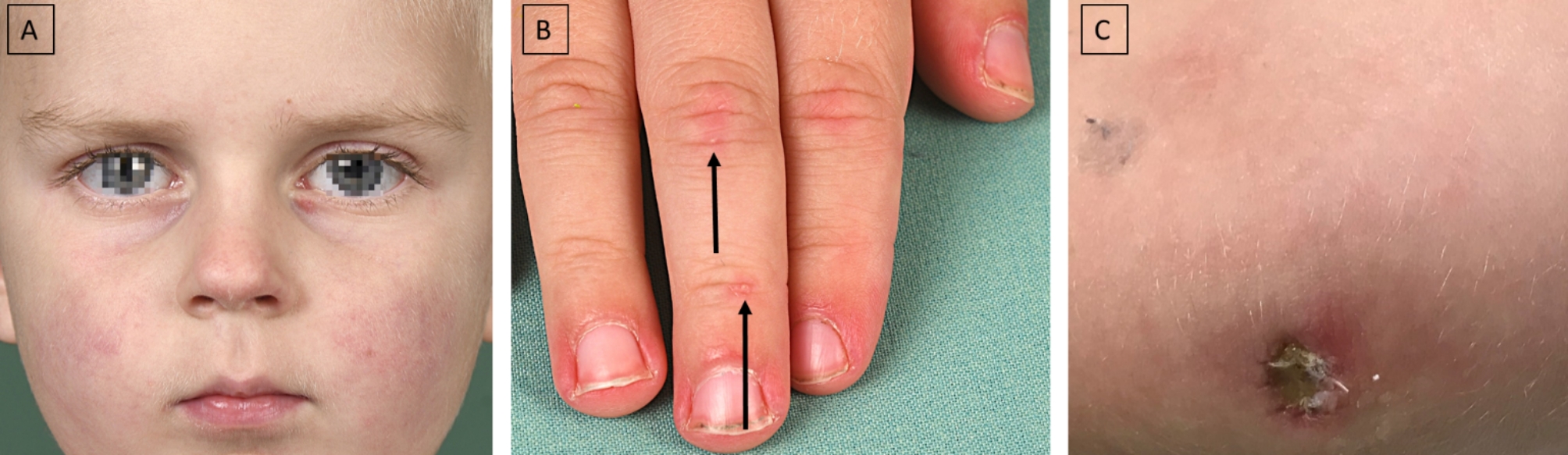
**A**: Patchy, pale erythema on both cheeks and subtle livid discoloration of eyelids. **B**: Periungual erythema, thickening of the nail fold, and incipient Gottron papules on the finger extensor sides. **C**: Livid macula with superimposed scaly plaque over ulceration on the right elbow. Symmetrical lesions were seen on the left elbow

A previously healthy 4-year-old boy was referred to our clinic suffering from acute respiratory insufficiency with oxygen demand. The parents observed reduced exercise tolerance, fatigue, and increased effort when climbing stairs over the past 4 months. More recently, arthritis in the area of the PIP and MCP joints of both hands, the knee and elbow joints on both sides, as well as skin changes with livid discolorations on both knees and elbows, the cheeks as well as the eyelids were noticed (see Fig. 1).

Under the suspicion of juvenile idiopathic arthritis with vasculitic component, a therapy with prednisolone (1 mg/kg), methotrexate (12,5 mg/m^2^) and adalimumab (20 mg) after failure of achieving a response had been initiated in the external clinic. The family history was unremarkable except for psoriatic arthritis in the child’s father.

**Fig. 2:**
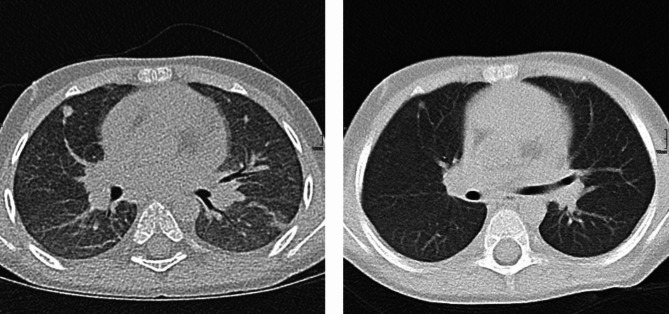
CT thorax before (left, September 2021) and after (right, November 2021) therapy initiation with prednisolone, intravenous immunoglobulins, cotrimoxazole and ruxolitinib. Left: pleural round spot between middle lobe and upper lobe; ventral milk glass opacities. Right: Remission of milk glass opacity in all lobes of the lung with single patchy residuals in the middle lobe and basal upper lobe on the right

**Fig. 3:**
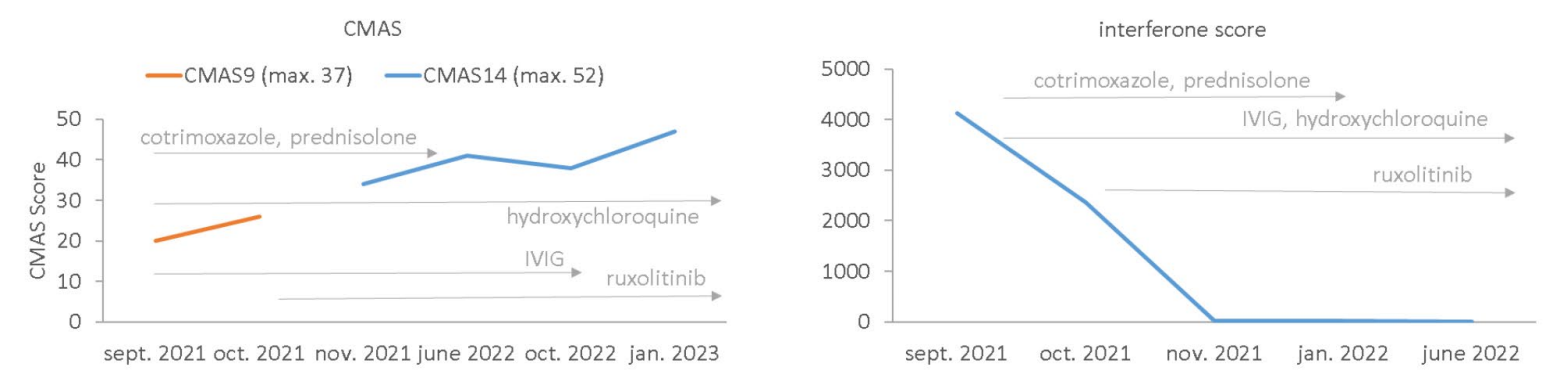
Improvement in CMAS score and decrease of interferone score during treatment. Starting from initial diagnosis in september 2021, this picture reveals the constant improvement in the childhood myositis assessment scale (CMAS) as well as the constant decrease of interferone score in our patient. The different treatments of the patient are displayed in light grey. For detailed information about the measurement of the interferone score, please see the supplementary material

Respiratory aggravation with cough and subfebrile temperatures as well as an ulcerating lesion in the area of the right elbow led to acute hospitalization of the boy in an external clinic. Under suspicion of bilateral pneumonia, calculated antibiotic therapy with ampicillin/sulbactam was started. Due to the lack of clinical improvement, a computed tomography (CT) of the thorax was performed, which revealed the picture of interstitial pneumonitis (see Fig. 2). Under the tentative diagnosis of MTX-induced pneumonitis, the patient was transferred to our clinic. On admission, we saw a 4-year-old boy in reduced general condition with tachydyspnea and auscultatory bilateral moist rale. Inspection of the skin revealed heliotrope discoloration of the eyelids, patchy-livid macules on the cheeks, scaly plaques with ulceration on both elbows, Gottron papules on the extensor sides of the fingers, marked erythema on both palmar sides with fine scaling, and livid-brownish macules over the extensor sides of both knees (cutaneous dermatomyositis disease area and severity index [cDASI]: activity 14, damage 2) (see Fig. 1). Clinically, the patient also showed a trunk-emphasized muscle weakness with a childhood myositis assessment scale (cMAS9) score of 9 out of 37 (see Fig. 3). The patient did not show clinical findings on his nail folds; a capillaroscopy was unremarkable.

Muscle enzymes were only slightly elevated (total CK activity 0.26 µmol/(s*L) [ref. range: <3.17 µmol/(s*L)], aldolase 0.22 µmol/(s*L) [ref. range: <0.2 µmol/(s*L)], LDH 5.37 µmol/(s*L) [Ref.-range: 2.0–5.0 µmol/(s*L)], AST 0.54 µmol/(s*L) [Ref.-range: <0.88 µmol/(s*L)]). A striking feature was a strongly increased interferon signature (4124 [Ref.-range: <12.49]) (see Fig. 3) and positive myositis-specific MDA5 alongside myositis-associated Ro52 autoantibodies (for detailed information about the measurement of the interferone signature, please see the supplementary material). The antinuclear autoantibodies (ANA) were positive (1:160). Both muscle sonography and MRI examination of the lower extremity and pelvis showed a picture of hypotrophic musculature without typical signs of myositis. In synopsis of the clinical and serological findings, we diagnosed hypomyopathic MDA5-positive jDM with calcinosis cutis in the elbow region and high-grade suspicion of interstitial lung involvement. Diagnostic bronchoscopy with bronchoalveolar lavage (BAL) revealed a lymphocytic infiltrate on histopathology, consistent with pulmonary involvement in jDM. However, Pneumocystis jirovecii was detected from the BAL, so the pulmonary changes including the histopathological findings were differentially diagnosed as PCJ pneumonia and intravenous antibiotic therapy with cotrimoxazole was started. In addition, we administered IVIG, continued therapy with prednisolone at 1 mg/kg bodyweight and supplemented therapy with hydroxychloroquine 200 mg/day. Further therapy with topical class III corticosteroid mometasone fuorate was applied on all cutaneous lesions except the face, which was changed to topical 0.1% tacrolimus ointment during the course of therapy. As pulmonary involvement in severe MDA5-positive jDM is a feared complication and could not be excluded despite the positive PCJ detection, further therapeutic procedure and therapy options such as cyclophosphamide, rituximab or JAK inhibition were discussed. Taking into account the highly elevated interferon signature (4124 [Ref.-range: <12.49]) and positive case reports in patients with jDM [14–16], we considered an off-label therapy trial with the JAK inhibitor ruxolitinib (since October 2021, 2 × 5 mg/day).

Following initiation of this therapy, continued improvement especially in respiratory symptoms occurred. CT thorax during the course showed an almost complete remission of findings with still minor interstitial drawing proliferation (see Fig. 2). Under the aforementioned therapeutic measures, there was a clear response with significant improvement in CMAS (CMAS at admission: 20/52 points; CMAS January 2023 47/52 points) (see Fig. 3). Encouragingly, the ulceration in the elbow region improved constantly. The lesions showed regular wound healing. Except for minor remaining scarring, the skin lesions completely resolved without relapse or clinical signs of calcinosis. Therapy with oral glucocorticoids was continuously tapered and finally stopped after six months. IVIGs were also stopped after 13 months of therapy.

As of today, the boy is currently in a sustained stable remission.

## Discussion and conclusion

In summary, this case report shows that hypomyopathic juvenile dermatomyositis should be considered in the presence of typical skin changes despite the absence of muscle enzyme elevation or radiological evidence. Importantly, skin involvement may be accompanied by mild barely visible bluish erythema as seen on cheek and ears in this case. Therefore, careful examination of the typically affected areas on extensor sites of arms, knees and thighs is required. As our case proofs, a patient with juvenile dermatomyositis may also present with arthritis due to the systemic manifestation of the disease [17].

In the therapy of jDM, the aim should be to control inflammation while restoring muscle strength and preventing long-term sequelae [12]. In this regard, validated measurement tools can objectify disease activity and provide guidance in therapy evaluation (e.g., muscle strength and function [CMAS, MMT8], cutaneous measurement tools [CAT], and composite scores [DAS or MDAAT]) [1].

Depending on disease severity, an individual therapy strategy should be used. In addition to standard therapies, further treatment escalations using rituximab, TNF-alpha inhibitors, or cyclophosphamide should be discussed [1, 12, 13]. Because of the high association between MDA5-autoantibodies and Pneumocystis jirovecii (PCJ) pneumonia, PCJ-prophylaxis should be considered in this risk constellation [18]. The treatment of our patient with cotrimoxazole may have contributed to the improvement of the respiratory symptoms. However, it is not possible to separate exactly what influence the treatment of the infection and the underlying disease had.

Moreover, in severe MDA5-positive juvenile dermatomyositis with calcinosis cutis as well as interstitial lung disease, some case reports describe that therapy extension with a JAK inhibitor may be a helpful therapeutic alternative to other immunosuppressive approaches such as rituximab or cyclophosphamide [14–16]. Further evidence is currently missing [14–16]. In patients with juvenile dermatomyositis, calcinosis cutis is a difficult-to-treat complication that can cause long-term mobility limitations in affected individuals [19]. The reported incidence varies between 23 and 70% [20, 21]. Early and aggressive treatment of jDM appears to reduce the likelihood of developing calcinosis cutis [22]. Randomized-controlled studies on the therapeutic management of calcinosis cutis are currently pending. However, some case reports describe a positive effect of JAK inhibition in jDM with calcinosis cutis [16, 23, 24]. The therapeutic response is probably due to a combination of reduced interferon response and anti-inflammatory effects through the reduction of proinflammatory cytokines [25, 26]. Accordingly, the patient in our case report showed a strong decrease in interferone signature after JAK inhibition (see Fig. 3).

Currently, the role of JAK inhibitors in jDM has not been conclusively established and requires individual clinical consideration due to their off-label use and side effect profile.

### Electronic supplementary material

Below is the link to the electronic supplementary material.

Supplementary Material 1

Supplementary Material 2

## Data Availability

Data sharing is not applicable to this article as no datasets were generated or analyzed during the current study.

## Abbreviations

jDM: Juvenile dermatomyositis
TIF1: Anti-transcription intermediary factor 1
NXP2: Nuclear matrix protein 2
SSA/SSB: Sjögren’s syndrome type A/B
Sm: Smith-antibody
MDA5: Melanoma differentiation-associated protein 5
JAK: Janus kinase
IVIG: Intravenous immunglobulins
cDMARD: Conventional disease-modifying antirheumatic drugs
TNF: Tumor nekrosis factor
PIP: Proximal interphalangeal
MCP: Metacarpophalangeal
CT: Computed tomography
MTX: Methotrexate
cDASI: Cutaneous dermatomyositis disease area and severity index
cMAS: Childhood myositis assessment scale
CK: Creatin kinase
LDH: Lactate dehydrogenase
AST: Aspartate aminotransferase
ANA: Antinuclear autoantibodies
MRI: Magnetic resonance imaging
BAL: Bronchoalveolar lavage
PCJ: Pneumocystis jirovecii
