# Supplementary material for: Rapid and sustained response to JAK inhibition in a child with severe MDA5 + juvenile dermatomyositis
Source: Pediatr Rheumatol Online J. 2023 Sep 19;21:104. doi: 10.1186/s12969-023-00894-9 (PMC10507825; doi:10.1186/s12969-023-00894-9)
Supplement: Supplementary file 1 — Supplementary Material 1 [file 12969_2023_894_MOESM1_ESM.docx]

Supplement to “Rapid and sustained response to JAK inhibition in a child with severe

MDA5+ juvenile dermatomyositis“

**Measurement of Interferon Signature**

Total RNA was extracted from PBMCs using the RNeasyMini Kit (Qiagen) followed by DNase I digestion. Gene expression was determined by quantitative real-time RT-PCR using Taqman Universal PCRMaster Mix (Applied Biosystems) on an ABI7300 and normalized to the expression of glyceraldehyde-3-phosphate dehydrogenase and hypoxanthine phosphoribosyltransferase 1 (Hs02800695_m1). For calibration, a calibrator cDNA was included in each assay. Target genes were analyzed using predesigned TaqMan probes for IFI44 (Hs00951349_m1), IFI44L (Hs00915292_m1), IFIT1 (Hs01675197_m1), ISG15 (Hs01921425_s1), RSAD2 (Hs01057264_m1), and SIGLEC1 (Hs00988063_m1). Oligonucleotides used for quantitative RT-PCR of GAPDH were for-GAAGGTGAAGGTCGGAGTC, rev-GAAGATGGTGATGGGATTTC, and FAM-CAAGCTTCCCGTTCTCAGCC-TAMRA. The IFN score was calculated as previously described (Wolf C et al. JACI 2020).
