# Supplementary material for: Rapid and sustained response to JAK inhibition in a child with severe MDA5 + juvenile dermatomyositis
Source: Pediatr Rheumatol Online J. 2023 Sep 19;21:104. doi: 10.1186/s12969-023-00894-9 (PMC10507825; doi:10.1186/s12969-023-00894-9)
Supplement: Supplementary file 2 — Supplementary Material 2 [file 12969_2023_894_MOESM2_ESM.docx]

Response to reviewers

Dear Dr. Spencer,

we would like to sincerely thank you for your review and processing of our manuscript. We are pleased that our article is of interest for the readership of your esteemed journal. Please find enclosed our response to the reviewers and the revised version of our manuscript (track-change).

Yours sincerely

Timmy Strauss on behalf of the co-authors

Reviewer #1:

*Thank you very much for your comments which we addressed carefully.*

1. The definition is somewhat unclear, is it hypomyopathic, amyopathic, clinical amyopathic? Please cite and refer to definitions in the literature, and/or define it in the introduction.

*Response from authors: thank you for the question which helped us to clarify the description of our case. We define our case as “hypomyopathic” due to the mild trunk-emphasized muscle weakness, only slightly elevated muscle enzymes and missing radiological signs of myositis. The new chapter in the case report now reads: “In synopsis of the clinical and serological findings, we diagnosed ~~amyopathic or~~ hypomyopathic MDA5-positive jDM with calcinosis cutis in the elbow region and high-grade suspicion of interstitial lung involvement“.*

*We further added a definition of the hypomyopathic dermatomyositis and a referring citation in the introduction. The new chapter now reads: “Sontheimer et al. define hypomyopathic dermatomyositis as “dermatomyositis-specific skin disease and no clinical evidence of muscle disease (i.e., weakness) that are found to have subclinical evidence of myositis upon laboratory, electrophysiological and/or radiological evaluation” (1).*

2. I did not find a capillaroscopy. Please report if this was done or not or if there were clinical findings.

*Response from authors: The patient did not show clinical findings on his nail folds; a capillaroscopy was unremarkable. We added the information to our case report.*

3. What about the arthritis, was it initially confirmed by ultrasound? Was it arthritis or just swelling as you write? Is arthritis usual in hyop/amyopathic JDM? Please report data from the literature

*Response from authors: Thank you for your question. Clinically, the external clinic described a polyarthritis with joint swelling and pain. Dermatomyositis as a systemic disorder may also cause an arthritis (2). An ultrasound was not performed. We changed the discussion section accordingly: “As our case proofs, a patient with juvenile dermatomyositis may also present with arthritis due to the systemic manifestation of the disease.”*

4. What type of interferone did you measure?

*Response from authors: Thank you very much. We now added a section about the measurement of the interferone signature in the supplementary material and referred to it in the manuscript.*

5. MRI without typical signs (please describe more in detail or show picture), was fasciitis present?

*Response from authors: The MRI showed an unspecific circumscribed subcutaneous edema and no fasciitis. Hypotrophic musculature could be confirmed.*

6. Please mention which damage manifestations (score 2) resolved completely?

*Response from authors: The damage manifestation of calcinosis cutis on both elbows resolved completely. The according paragraph can be found from line 164-166 in the manuscript.*

7. Why was it not the infection with PCJ that resolved? Explain with the cited studies referring the association between MDA5 antibodies and PCJ. What is the usual course of ILD depending on treatment, please report literature data. How often is ILD reported in amyopathic JDM?

*Response from authors: Thank you very much for the questions which address an interesting point of our manuscript. The boy in our case report presented with severe clinical signs of respiratory distress. The computed tomography (CT) of the thorax revealed the picture of interstitial pneumonitis which might lead to the diagnosis of interstitial lung involvement in juvenile dermatomyositis. On the other hand, Pneumocystis jirovecii was detected from the BAL, so the pulmonary changes were differentially diagnosed as PCJ pneumonia. Due to the severity of the disease, we immediately started the combined treatment with intravenous antibiotic therapy (cotrimoxazole) and the mentioned jDM therapy with IVIG, prednisolone and JAKi. The combination of the different therapy options led to complete clinical remission. We therefore believe that our case is helpful to draw attention to the challenging clinical differentiation between interstitial lung disease in jDM and PCJ-pneumonia in association with MDA5 autoantibodies.*

*A review from 2022 (3) reported that up to 80% of patients with clinically amyopathic dermatomyositis can develop ILD. If MDA5 autoantibodies are present, the association with ILD is even higher (up to 95%). The ILD in MDA5 autoantibody positive jDM is critical due to its treatment refractory course and high mortality rates (6-month mortality up to 50%).*

*We added this clarification to the manuscript.*

Reviewer #2: Thank you for the opportunity to review this work

*Thank you very much for your comments which we addressed carefully.*

1. Infant would not normally be used for a child of 4 years (title)

*Response from authors: We agree and changed the title to “child”.*

2. There are some language issues which impact readability and at times make meaning incorrect or ambiguous.

*Response from authors: Thank you very much for the comment. A native speaker has gone through the manuscript again. If you still find language issues, please let us know.*

3. Page 5. Meaning of "score of 9 out of 20/37" when discussing CMAS is unclear. Note that CMAS is not considered valid under 5, although still reasonable to report

*Response from authors: for the first assessment, we used the CMAS-score with 9 items (CMAS9) with a maximum possible score of 37. For the following assessments we used the complete CMAS-score with 14 items. The new sentence now reads: “Clinically, the patient also showed a trunk-emphasized muscle weakness with a childhood myositis assessment scale (cMAS9) score of 9 out of 37.”*

4. Page 5. Meaning of s*L is not clear

*Response from authors: the “s*l” stands for “second*liter” and is part of the international SI-unit system. We provide reference ranges for a better comparability.*

5. Page 6. ASAT would usually be abbreviated AST

*Response from authors: Thank you for the comment. We changed the abbreviation accordingly.*

6. Page 6. Interferon signature is not a standardized test-additional detail needed about products assessed and methods

*Response from authors: Thank you very much. We now added a section about the measurement of the interferone signature in the supplementary material and referred to it in the manuscript.*

7. Page 6. Calling this patient amyopathic when CMAS score is 20 (or lower) and there is clinical weakness would not be considered accurate.

*Response from authors: Thank you for pointing that out which helped us to clarify the description of our case. We define our case as “hypomyopathic” due to the mild trunk-emphasized muscle weakness, only slightly elevated muscle enzymes and missing radiological signs of myositis. The new chapter in the case report now reads: “In synopsis of the clinical and serological findings, we diagnosed ~~amyopathic or~~ hypomyopathic MDA5-positive jDM with calcinosis cutis in the elbow region and high-grade suspicion of interstitial lung involvement“.*

*We further added a definition of the hypomyopathic dermatomyositis and a referring citation in the introduction. The new chapter now reads: “Sontheimer et al define hypomyopathic dermatomyositis as “dermatomyositis-specific skin disease and no clinical evidence of muscle disease (i.e., weakness) that are found to have subclinical evidence of myositis upon laboratory, electrophysiological and/or radiological evaluation” (1)*

8. Page 6-regurgitation is definitely the wrong words. Other language errors scattered through document

*Response from authors:* We are sorry for the mistake and changed the word to “remission”.

9. Page 7. "age-physiological desire and extent of movement" meaning unclear

*Response from authors: Thank you for your comment, we deleted this sentence due to its inter-individual variety.*

10. Page 7. Given the above comment about considering this patient to be amyopathic/hypomyopathic, the opening sentence of the discussion is not relevant. Consider modifying to address the delay in diagnosis due to patient being considered JIA at the referring institution

*Response from authors: Thank you very much, with this sentence we tried to emphasize the problem of missing muscle enzymes or radiological evidence in the diagnosis of jDM. We further added a sentence regarding the initial arthritis in our patient. It now reads:*

*As our case proofs, a patient with juvenile dermatomyositis may also present with arthritis due to the systemic manifestation of the disease.” (2)*

11. Page 7. "treat to target" strategies have neither been defined or evaluated in JDM.

*Response from authors: Thank you for the comment, we changed the “treat to target” to “individual therapy strategy”.*

12. Page 7-8. The statement that "therapy extension with a JAK inhibitor represents an effective therapeutic alternative to other immunosuppressive approaches such as rituximab or cyclophosphamide" is inappropriate. There is no evidence to show this, and this work cannot provide this evidence. It is possible it is true, but making this a statement of fact is incorrect.

*Response from authors: We changed the sentence accordingly, it now reads: “, some case reports describe that therapy extension with a JAK inhibitor may be a helpful therapeutic alternative to other immunosuppressive approaches such as rituximab or cyclophosphamide. Further evidence is currently missing.”*

13. Page 7-8. The discussion/conclusion appears to have paragraphs addressing several concepts and points. It should be broken up by additional paragraphs to make it easier to follow.

*Response from authors: We now added additional paragraphs in the discussion to improve readability.*

14. I note that the authors provide a statement that consent was obtained from the family to publish an identifiable photograph.

*Response from authors: The patient's parents gave their written consent to the publication of the case report and included patient photographs on Jan. 24, 2023.*

Reviewer #3: O artigo precisa ser revisado pois não está clara a importância do tratamento com iJAK neste caso. Faltam informações sobre os tratamentos realizados previamente quando foi feito o diagnóstico de JIA que pode ter modificado o quadro clínico e laboratorial. Há pouca informação sobre qual iJAK foi usada , qual dose, por quanto tempo e a sequencia cos parâmetros de avaliação. 
O paciente tem história de fraqueza muscular, CMAS com baixa pontuação o que não é compatível com JDM amiopática.

(engl. Translation: The article needs to be revised as it is not clear the importance of iJAK treatment in this case. There is a lack of information about the treatments previously performed when the diagnosis of JIA was made, which may have modified the clinical and laboratory picture. There is little information about which iJAK was used, what dose, for how long and the sequence of evaluation parameters.

The patient has a history of muscle weakness, CMAS with low score which is not compatible with amyopathic JDM.)

*Response from authors: Thank you very much for your review. We tried to translate your comments carefully and hope to address your questions correctly.*

1. “There is a lack of information about the treatments previously performed when the diagnosis of JIA was made, which may have modified the clinical and laboratory picture.”

*Response from authors: Previous treatment is provided on page 6 line 113 – 115.We provided further information on the dosage used.*

2. “There is little information about which iJAK was used, what dose, for how long and the sequence of evaluation parameters.”

*Response from authors: Thank you very much for your comments which helped us to clarify the use of ruxolitinib. We now provide information about dosage and duration of the JAK-inhibitor therapy. We already provided information about the improvement in CMAS score in the following sentence: Under the aforementioned therapeutic measures, there was a clear response with significant improvement in CMAS (CMAS at admission: 20/52 points; CMAS January 2023 47/52 points)”.*

3. “The patient has a history of muscle weakness, CMAS with low score which is not compatible with amyopathic JDM.”

*Response from authors: thank you for the comment which helped us to clarify the description of our case. We define our case as “hypomyopathic” due to the mild trunk-emphasized muscle weakness, only slightly elevated muscle enzymes and missing radiological signs of myositis. The new chapter in the case report now reads: “In synopsis of the clinical and serological findings, we diagnosed ~~amyopathic or~~ hypomyopathic MDA5-positive jDM with calcinosis cutis in the elbow region and high-grade suspicion of interstitial lung involvement“.*

*We further added a definition of the hypomyopathic dermatomyositis and a referring citation in the introduction. The new chapter now reads: “Sontheimer et al. define hypomyopathic dermatomyositis as “dermatomyositis-specific skin disease and no clinical evidence of muscle disease (i.e., weakness) that are found to have subclinical evidence of myositis upon laboratory, electrophysiological and/or radiological evaluation” (1)*

4. Title: Paciente de 4 anos sendo considerado infant?

*Response from authors: We are sorry for the wrong labeling and changed the title to “child”.*

5. Abbreviations
ASAT

*Response from authors: Thank you very much for the comment. We changed the abbreviation accordingly.*

Background necessita revisãoi (transl. “needs revision”)
L72
L79-81

Case presentation - necessita revisão
L102
L105
L109-L119
L135
L142
145
L149
L154

Discussion and conclusion - necessita revisão
L58-L159
L166-167
L169-172
L175-176
L185

*Response from authors: We apologize but we do not know what you exactly mean when you write down the line numbers. May we please ask you to clarify the points that need a revision? We thank you very much for your clarification.*

Figure 3
Falta legenda e marcação da das datas

*Response from authors: We extended the figure legend. It now reads: “improvement in CMAS score and decrease of interferone score during treatment. Starting from initial diagnosis in September 2021, this picture reveals the constant improvement in the childhood myositis assessment scale (CMAS) as well as the constant decrease of interferone score in our patient. The different treatments of the patients are displayed in light grey. For detailed information about the measurement of the interferone score, please see the supplementary material.”*

Reviewer #4: The authors reported a case of MDA5+ JDM treated with a JAK inhibitor. 
Treatment of JDM with JAKi is still rare. Thus, this is a valuable case report. However, more than 10 case reports/series have been reported. Thus, it is not so novel.
In addition, the title of this case report states that it is an infant case, but it is a 4-year-old case, a title that could be misinterpreted as a novelty.

*Response from authors: Thank you very much for reviewing our manuscript. We believe that growing evidence may help clinicians to draw attention to this rare disease. Our case report describes a severe course of MDA5 positive jDM and positive response to JAK-inhibition. Thus, it may be of interest for clinicians with comparable cases.*

*We changed the title from “infant” to “child” and apologize for the mistake.*

Reviewer #5: Hi dear colleague 
JDM is a rare and important disease in pediatric rheumatology field. Treatment modalities are life saving and may be difficult. Your patient is an interesting and useful case report. 
Best wishes.

*Response from authors: Thank you very much for reviewing our manuscript.*

Reviewer #6: In this case report, Strauss et al. present the case of a 4-year-old patient with anti-MDA5 positive JDM and evidence of ILD who responded well to management with JAK inhibition. The case is an interesting one, though not entirely novel, does add support to a growing body of literature that suggest the utility of JAK inhibition in JDM.

*Thank you very much for your comments which we addressed carefully.*

Comments: 
1. Minor edit: Page 4, line 77: anti-MDA5 antibodies are listed, but MDA5 is not spelled out until line 79.

*Response from authors: Due to the readability of the sentence, we refused to spell out all the mentioned autoantibodies. We nevertheless updated our abbreviation list at the beginning of our manuscript. The clarification of “MDA5”, which is the crucial autoantibody for our case, can be found in the following sentence in line 83.*

2. Minor edit: Page 6, line 145: "regurgitation" may not be the correct word. Consider almost complete "resolution" Would need to edit figure legend as well.

*Response from authors:* we are sorry for the mistake and changed the word to “remission”.

3. Would also specify the timing of first CT and follow up CT to allow the reader to understand the duration of time between scans. Also, what is the role that treating PJP had in the overall clinical and radiographic improvement? The CT legend states that images were before and after steroids, IVIG and cotrimoxazole, with no mention of the JAK inhibition. Would include timelines of treatment and assessments. Would also include a discussion about the PJP management (potentially also playing a role in clinical response) in the discussion.

*Response from authors: Thank you very much for your comment. We added the time points of the different CT scans in the figure caption. We further added the JAK-inhibitor ruxolitinib to the figure caption. Due to the severity of the disease in our patient, we started the different treatments with no long time-gap in between them. We therefore saw an “overall effect” of the treatments which resulted in a complete remission. We added a paragraph to the discussion section: “the treatment of our patient with cotrimoxazole may have contributed to the improvement of the respiratory symptoms.”*

4. How extensive was the original calcinosis? Was imaging done to assess the extent of calcinosis? What degree of improvement did the patient have with respect to the calcinosis? Details surrounding the calcinosis would also likely be of great interest.

*Response from authors: Thank you very much for your comment. The calcinosis was present as scaly plaques with ulceration in both elbows. We did not assess radiological imaging because the calcinosis lesions were clinically impressive. The calcinosis improved constantly after initiation of therapy. The skin lesions completely resolved. We provided this information in the “case presentation”-section starting from line 164.*

**References**

1.	Sontheimer RD. The management of dermatomyositis: current treatment options. Expert opinion on pharmacotherapy. 2004;5(5):1083-99.

2.	Callen JP. Dermatomyositis. The Lancet. 2000;355(9197):53-7.

3.	McPherson M, Economidou S, Liampas A, Zis P, Parperis K, editors. Management of MDA-5 antibody positive clinically amyopathic dermatomyositis associated interstitial lung disease: A systematic review. Seminars in Arthritis and Rheumatism; 2022: Elsevier.
